# Assessing the molecular divergence between *Anopheles *(*Kerteszia*) *cruzii *populations from Brazil using the *timeless *gene: further evidence of a species complex

**DOI:** 10.1186/1475-2875-8-60

**Published:** 2009-04-09

**Authors:** Luísa DP Rona, Carlos J Carvalho-Pinto, Carla Gentile, Edmundo C Grisard, Alexandre A Peixoto

**Affiliations:** 1 Laboratório de Biologia Molecular de Insetos, Instituto Oswaldo Cruz, FIOCRUZ, Av. Brasil 4365, Rio de Janeiro 21045-900, RJ, Brazil; 2 Departamento de Microbiologia e Parasitologia, CCB, Universidade Federal de Santa Catarina, Florianópolis 88040-970, SC, Brazil; 3 School of Biological and Chemical Sciences, Queen Mary University of London, 327 Mile End Road, London, E1 4NS, UK

## Abstract

**Background:**

*Anopheles *(*Kerteszia) cruzii *was the most important vector of human malaria in southern Brazil between 1930–1960. Nowadays it is still considered an important *Plasmodium *spp. vector in southern and south-eastern Brazil, incriminated for oligosymptomatic malaria. Previous studies based on the analysis of *X *chromosome banding patterns and inversion frequencies in *An. cruzii *populations from these areas have suggested the occurrence of three sibling species. In contrast, two genetically distinct groups among *An. cruzii *populations from south/south-east and north-east Brazil have been revealed by isoenzyme analysis. Therefore, *An. cruzii *remains unclear.

**Methods:**

In this study, a partial sequence of the *timeless *gene (~400 bp), a locus involved in the control of circadian rhythms, was used as a molecular marker to assess the genetic differentiation between *An. cruzii *populations from six geographically distinct areas of Brazil.

**Results:**

The *timeless *gene revealed that *An. cruzii *from Itaparica Island, Bahia State (north-east Brazil), constitutes a highly differentiated group compared with the other five populations from south and south-east Brazil. In addition, significant genetic differences were also observed among some of the latter populations.

**Conclusion:**

Analysis of the genetic differentiation in the *timeless *gene among *An. cruzii *populations from different areas of Brazil indicated that this malaria vector is a complex of at least two cryptic species. The data also suggest that further work might support the occurrence of other siblings within this complex in Brazil.

## Background

*Anopheles cruzii *is one of the few mosquito species belonging to the subgenus *Kerteszia*. Immature stages of this species are found associated with water trapped in the interfoliar space of plants from the Bromeliaceae family, which are abundant in the Brazilian Atlantic forest [1-3]. Accordingly, the distribution of these bromeliad-breeding mosquitoes is restricted to the Atlantic forest, which stretches from the coast of Rio Grande do Sul State (southern Brazil) to Sergipe State (north-eastern Brazil) [4,5].

The adults are found in a variety of habitats, from sea level in coastal areas to the mountains. Females are strongly anthropophilic and preferably bite during the evening [2,6,7], perhaps biting more than one host to complete egg maturation, which is epidemiologically relevant for malaria transmission [8-10].

Between 1930 and 1960, *An. cruzii *together with *Anopheles bellator *and *Anopheles homunculus*, which also belong to *Kerteszia*, were considered the main vectors of malaria when the disease was endemic in southern Brazil. Vector control measures have significantly reduced or even interrupted malaria transmission in some areas, but eradication of the pathogen was not achieved and *An. cruzii *is still responsible for several oligosymptomatic malaria cases in southern and south-eastern Brazil.

The Amazon region is highly endemic for human malaria, caused by *Plasmodium vivax *and *Plasmodium falciparum*, and imported cases are frequently reported in different states due to emigration from this region [11,12]. However, several autochthonous cases were reported in a study in Santa Catarina State, southern Brazil [12]. In the states of São Paulo and Rio de Janeiro, as well as in the state of Bahia, where *An. cruzii *and *Anopheles (Nyssorhynchus) *spp. are considered the main vectors of the disease, respectively [3,7,13,14], several imported and autochthonous cases of malaria are reported every year in the Atlantic forest region [15]. Reinforcing the epidemiological importance of *An. cruzii *as a malaria vector in south-east Brazil, another recent study in Espírito Santo State, including the locality of Santa Teresa, suggested that this species is the potential vector of recent autochthonous cases of malaria in this state [16].

*Anopheles cruzii *is also a natural vector of simian malaria in Rio de Janeiro and São Paulo States [17]. Studies on seasonal and vertical distribution of *An. cruzii *in coastal São Paulo State demonstrated high vertical mobility from ground level to tree tops, with significantly more activity in the uppermost branch layer of the forest [18]. This behaviour could be responsible for human infection by simian *Plasmodium *species [19,20].

Epidemiological surveillance and the use of control measures are required to avoid the expansion or introduction of malaria in areas where vector species are abundant and susceptible humans are present. Thus, assessment of the epidemiological status of such localities as well as knowledge concerning the biology, behaviour and the genetic characteristics of the vector species are relevant to prevent the occurrence of outbreaks or to lead control strategies, especially in formerly endemic areas.

Despite its epidemiological importance, there are only a few population genetic studies of *An. cruzii *[18,21], and its taxonomic status is unclear. *Anopheles cruzii *is polymorphic for chromosome rearrangements. Differences in inversions frequencies, and *X *chromosome banding patterns from south-eastern and southern Brazil, have suggested the existence of three sibling species [21-24]. On the other hand, isoenzymes indicated two genetically isolated groups, one from Bahia State (north-eastern Brazil), and the other from south-eastern and southern Brazil (Rio de Janeiro, São Paulo and Santa Catarina States) [25]. Finally, in a recent study based on sequence analysis of the second Internal Transcribed Spacer of the nuclear ribosomal DNA (ITS2), the authors found no conclusive evidence for sibling species among samples of *An. cruzii *from south-eastern and southern Brazilian localities [26].

The activity and feeding rhythms of insect vectors are very important to disease transmission. These patterns are controlled by endogenous circadian clocks, which are under genetic control [27]. Furthermore, clock genes are also involved in the control of mating rhythms that are potentially important in maintaining sexual isolation between closely related species [28,29].

The circadian rhythms of malaria vectors belonging to the subgenus *Kerteszia *were formerly studied by Pittendrigh [30] and, recently, these rhythms were also studied in *An. cruzii *[31]. The *timeless *gene is involved in the control of activity rhythms in *Drosophila *[27], and controls differences in mating rhythms between closely related *Drosophila *species [28].

In the present study, a fragment of ~400 bp of the *An. cruzii timeless *gene was used as a molecular marker to assess intraspecific variability and genetic divergence among six populations of *An. cruzii *captured in different locations within the geographic distribution range of this species in Brazil.

## Methods

### Mosquitoes

**Figure 1:**
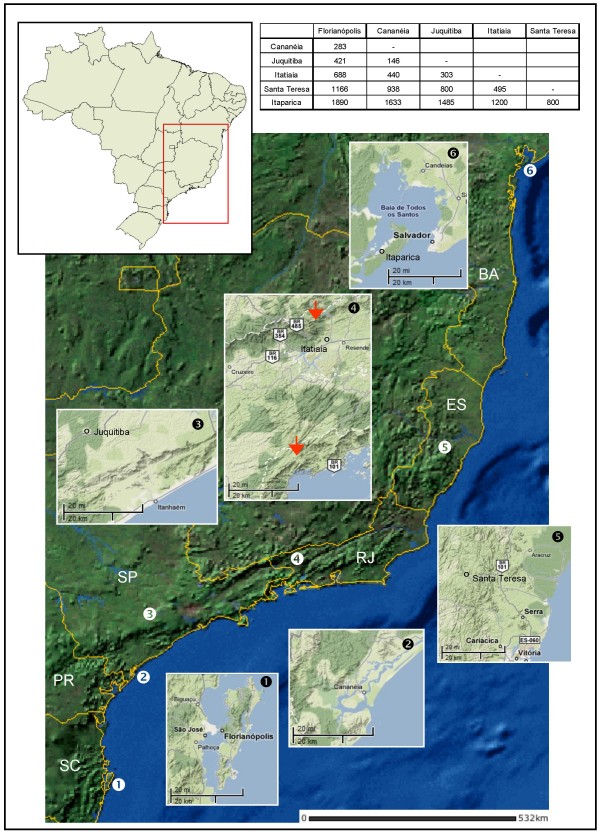
***Anopheles cruzii *populations**. Localities where the six Brazilian *An. cruzii *populations were collected. Values in table are approximated distances between localities in km. The red arrows on box 4 show the two mountain chains around Itatiaia. The upper arrow shows the Serra da Mantiqueira and the lower shows Serra do Mar mountain chains (Source: IBGE and Google Maps).

All mosquitoes used in this study were females captured at the following localities along the Brazilian Atlantic forest: Florianópolis, Santa Catarina State (SC) (27°31'S/48°30'W), Cananéia and Juquitiba, São Paulo State (SP) (25°01'S/47°55'W and 23°57'S/47°03'W), Itatiaia, Rio de Janeiro State (RJ) (22°27'S/44°36'W), Santa Teresa, Espírito Santo State (ES) (19°56'S/40°35'W) and Itaparica Island (Jaguaripe), Bahia State (BA) (13°05'S/38°48'W) (Figure 1). All mosquitoes were primarily identified on the basis of their morphology according to Consoli and Lourenço-de-Oliveira [5]. A total of 67 individuals (12 from Florianópolis, 12 from Cananéia, 11 from Juquitiba, 12 from Itatiaia, 6 from Santa Teresa and 14 from Itaparica, Bahia) were used for molecular assays.

### Isolation of the *An. cruzii *timeless gene sequence

**Figure 2:**
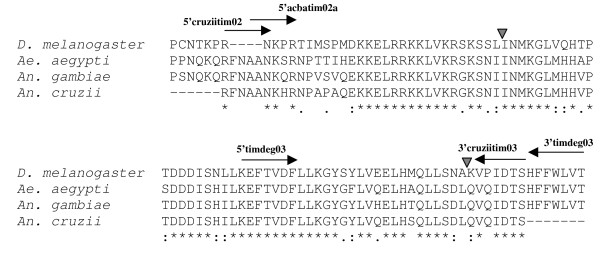
**Timeless protein multiple alignment and primer positions**. The putative fragment of *An. cruzii *TIMELESS deduced protein is aligned with *D. melanogaster*, *An. gambiae *and *Ae. aegypt *homologues. Arrows point to the approximated positions of the primers used in this study. The inverted triangles represent the positions of the two introns.

To design specific primers for the *An. cruzii timeless *gene sequence, genomic DNA was extracted from 10 females according to Jowett [32]. Initially, a pair of degenerated primers based on conserved regions of the TIMELESS proteins from *Drosophila melanogaster *and *Anopheles gambiae *named here 5'timdeg03 and 3'timdeg03 was used (Table 1; see also Figure 2) [33]. PCR was carried out with an Eppendorf Mastercycler^® ^thermocycler using the following conditions: 15 cycles at 94°C for 60 s, 50°C (decreasing 1°C/cycle) for 90 s and 72°C for 60 s, following 20 cycles of 94°C for 60 s, 50°C for 90 s and 72°C for 60 s. The products obtained were then purified and cloned in either Zero Blunt TOPO PCR cloning kit (Invitrogen) or pMOS *Blue *vector blunt-ended cloning kit (Amersham Biosciences). Sequencing of positive clones was carried out in an ABI Prism 377 or ABI Prism 3730 DNA sequencer at the Oswaldo Cruz Institute using the ABI Prism Big Dye Terminator Cycle Sequencing Ready Reaction kit (Applied Biosystems). The identity of the cloned fragments was determined by BlastX analysis using the GenBank [34]. To enlarge the *timeless *gene fragment in *An. cruzii*, a specific forward primer (5'darltim02a) based on a fragment of the *Anopheles darlingi timeless *gene (Gentile & Peixoto, unpublished) was used in combination with the specific reverse primer previously designed for *An. cruzii *(3'cruziitim03) in a PCR that amplified a fragment of ~450 bp. This 450 bp fragment from the *An. cruzii *genome was then purified, cloned and sequenced as above. After checking the sequence identity, two new specific forward primers named 5'cruziitim02 and 5'acbatim02a (Table 1 and Figure 2) were designed and in combination with the reverse primer 3'cruziitim03 allowed the amplification of a ~400 bp fragment of the *An. cruzii timeless *gene.

**Table 1:** Sequence of primers used to amplify the *timeless *gene fragments

Primers Name	Sequence of primers at 5' → 3'
5'timdeg03	AARGARTTYACNGTNGAYTT (forward)
3'timdeg03	GTNACNARCCARAARAARTG (reverse)
3'cruziitim03	GACGTATCGATCTGCACTT (reverse)
5'cruziitim02	CGCTTCAATGCCGCAAATA (forward)
5'acbatim02a	GCCGCAAATAAGCACCG (forward)

Degenerate and specific primers used to amplify the *timeless *gene fragments in all *Anopheles cruzii *populations.

### Interpopulational analysis of the *An. cruzii *timeless gene

Females were processed individually and genomic DNA was extracted as above [32]. PCR amplification was carried out for 35 cycles at 94°C for 30 s, 62°C for 60 s and 72°C for 90 s using the proofreading *Pfu *DNA polymerase (Biotools) and primers 5'acbatim02a or 5'cruziitim02 and 3'cruziitim03 (Table 1). Negative controls (no DNA added) were included in all amplification reactions and pre- and post-PCR procedures did not share equipment or reagents. After cloning the fragments obtained as above, at least eight clones of each mosquito were sequenced and two consensus sequences representing both alleles were generated. When only one haplotype was observed among the eight sequences the mosquito was considered a homozygote. The probability that a heterozygote will be mistakenly classified as a homozygote with this procedure is less than 1%. Five mosquitoes were classified as homozygotes in Itatiaia, none in Florianópolis and one in each of the other four populations. The sequences obtained in homozygote mosquitoes were duplicated prior to analysis. However, the population genetics analysis was also carried out without duplicating the homozygote sequences and the results were very similar.

### DNA sequence analysis

The *timeless *gene fragments were aligned using the GCG package (Wisconsin Package Version 10.2, Genetics Computer Group) and ClustalX software [35]. Analyses of the polymorphism and differentiation between populations were performed using DNASP4.0 [36] and P_RO_S_EQ _programs [37]. *F*_*ST *_was calculated as described by Hudson *et al *[38] and significance was evaluated by 1,000 random permutations. Phylogenetic analysis was carried out using MEGA 4.0 [39] using the default parameters.

## Results

### Isolation of *An. cruzii *timeless gene fragment

Different PCR schemes were tested to amplify a fragment of the *An. cruzii timeless *gene (see Methods). Figure 2 shows an alignment of the predicted amino acid sequence encoded by this fragment obtained from *An. cruzii *compared to the TIMELESS protein of other insect species (*D. melanogaster*, *Aedes aegypti *and *An. gambiae*). A fairly high degree of inter-specific similarity is observed, but the putative protein encoded by 5' end of this fragment is variable, presenting some amino acid changes among the species compared. Figure 2 also shows the approximate positions of the two introns that occur in this region of the gene, as well as the location of the primers used to amplify the fragment from *An. cruzii *used for the population genetics analysis described below.

### Molecular variation and divergence among *An. cruzii *populations

The geographic distribution of the six Brazilian populations of *An. cruzii *used in this study is shown at Figure 1. Initially, using the primers 5'cruziitim02 and 3'cruziitim03 (see Figure 2), a ~420 bp fragment of the *timeless *gene was amplified in all *An. cruzii *populations analyzed, with the exception of samples from Bahia State (Itaparica Island), which revealed a ~400 bp amplification product, indicating the existence of length variation among the studied populations. The sample from Bahia, however, displayed lower amplification in some cases using these primers, and so a new internal forward primer named 5'acbatim02a (Table 1) was designed based on the initial sequences obtained. Using this new primer in conjunction with 3'cruziitim03, a ~410 bp fragment of *timeless *gene was obtained for all *An. cruzii *populations from south and south-east Brazil and a ~390 bp from Bahia.

A total of 24 sequences from Florianópolis, 24 from Cananéia, 22 from Juquitiba, 24 from Itatiaia, 12 from Santa Teresa and 28 from Itaparica (Bahia State) populations were obtained. The sequences were submitted to GenBank (accession numbers: FJ408732 – FJ408865). A full alignment of all sequences is shown in Additional file 1. Most of the base substitutions were silent or occurred within the two introns, which show a number of indels. A few non-synonymous changes were also observed, causing seven amino acid differences among the sequences.

Table 2 shows the number of DNA sequences of each *An. cruzii *population studied (*n*) and the number of polymorphic sites (S). The values in parentheses were calculated using only coding regions of the *timeless *gene fragment. Based on the sequences, two measures of nucleotide diversity were calculated for each population: π, based on the average number of pair-wise differences and θ, based on the total number of mutations (Table 2). The population from Bahia was the least polymorphic, showing the lowest values of θ and π, as well as the smaller number of polymorphic sites (S). Table 2 also shows the results of Tajima [40] and Fu & Li [41] tests of natural selection, based on the total number of mutations of each population. In all cases, Tajima's *D *or Fu & Li's *D *and *F *statistics were non-significant (P > 0.10) indicating no deviations from neutrality.

**Table 2:** Polymorphisms of all *An. cruzii *populations

Population	*n*	S	θ	π	*D*_T_	*D*_FL_	*F*_FL_
Florianópolis	24	57 (17)	0.04258 (0.02322)	0.03018 (0.01483)	-1.00660 (-1.24295)	-0.62541 (-1.24456)	-0.87450 (-1.45349)
Cananéia	24	46 (12)	0.03334 (0.01665)	0.02677 (0.01021)	-0.64691 (-1.30282)	-0.47115 (-0.96989)	-0.61709 (-1.24756)
Juquitiba	22	52 (20)	0.03522 (0.02652)	0.03086 (0.02217)	-0.48955 (-0.51723)	-0.47485 (-0.34701)	-0.56076 (-0.46415)
Itatiaia	24	26 (12)	0.01864 (0.01665)	0.01829 (0.01825)	-0.00645 (0.40503)	-0.32168 (0.25917)	-0.25815 (0.35329)
Santa Teresa	12	35 (15)	0.03042 (0.02558)	0.02518 (0.02248)	-0.65598 (-0.41589)	-0.86793 (-0.58337)	-0.92570 (-0.61405)
Bahia	28	24 (9)	0.01661 (0.01099)	0.01035 (0.00571)	-1.31797 (-1.49603)	-0.83982 (-0.91433)	-1.16519 (-1.27249)

*n*, number of DNA sequences of each population; S, number of polymorphic (segregating) sites; *θ*, nucleotide diversity based on the total number of mutations (*Eta*); *π*, nucleotide diversity based on the average number of pair-wise differences; *D*_T_, Tajima's [40]*D*; *D*_FL_, Fu & Li's [41]*D *and *F*_FL_, Fu & Li's [41]*F*, based on the total number of mutations. In no cases were Tajima's *D*-values or Fu & Li's *D *and *F*-values significant (P > 0.10 in all cases). The values in parentheses were calculated using only coding regions of the *timeless *gene fragment.

Table 3 shows the pair-wise estimates of population differentiation (*F*_*ST*_) between all *An. cruzii *populations. In all cases the *F*_*ST *_values were significant, except for the comparison between Juquitiba and Santa Teresa when the coding regions of the *timeless *gene fragment were used. Very high *F*_*ST *_values were found between Bahia State and the others (0.8353 – 0.8761). The average number of nucleotide substitutions per site (*Dxy*) and the number of net nucleotide substitutions per site between populations (*Da*) are shown in Table 3. The distribution of the four mutually exclusive categories of segregating sites observed in each comparison, i.e. the number of polymorphisms exclusive for each population (*S1 *and *S2*), the number of shared polymorphisms (*Ss*) and the number of fixed differences (*Sf*) between populations are also included in Table 3. These polymorphic and fixed sites include some of the non-synonymous changes observed (see Table 4 for a detailed description).

**Table 3:** Genetic differentiation between all *An. cruzii *populations

Populations	*F*_*ST*_	*P*-value	*Dxy*	*Da*	*S*_*s*_	*S*_*f*_	*S*_*1*_	*S*_*2*_
1. Florianópolis × Cananéia	0.0548 (0.0622)	0.002 (0.003)	0.0308 (0.0136)	0.0017 (0.0008)	30 (7)	0 (0)	28 (11)	17 (6)
2. Juquitiba × Santa Teresa	0.0693 (0.0487)	0.040 (0.156)	0.0290 (0.0236)	0.0020 (0.0011)	21 (10)	0 (0)	26 (10)	11 (6)
3. Florianópolis × Juquitiba	0.0875 (0.1384)	0.000 (0.000)	0.0333 (0.0216)	0.0029 (0.0030)	21 (8)	0 (0)	37 (10)	26 (12)
4. Cananéia × Juquitiba	0.1077 (0.1849)	0.002 (0.000)	0.0322 (0.0201)	0.0035 (0.0037)	20 (5)	0 (0)	27 (8)	27 (15)
5. Florianópolis × Itatiaia	0.1450 (0.2078)	0.000 (0.000)	0.0293 (0.0213)	0.0042 (0.0044)	16 (7)	0 (0)	42 (11)	11 (6)
6. Florianópolis × Santa Teresa	0.1582 (0.2652)	0.000 (0.000)	0.0325 (0.0256)	0.0051 (0.0068)	14 (8)	0 (0)	44 (10)	18 (8)
7. Itatiaia × Santa Teresa	0.1837 (0.2414)	0.000 (0.000)	0.0265 (0.0273)	0.0049 (0.0066)	10 (6)	0 (0)	17 (7)	22 (10)
8. Juquitiba × Itatiaia	0.2030 (0.2078)	0.000 (0.000)	0.0310 (0.0258)	0.0063 (0.0054)	10 (6)	0 (0)	37 (14)	17 (7)
9. Cananéia × Santa Teresa	0.2154 (0.3152)	0.000 (0.000)	0.0328 (0.0243)	0.0071 (0.0076)	11 (4)	0 (0)	36 (9)	21 (12)
10. Cananéia × Itatiaia	0.2251 (0.2720)	0.000 (0.000)	0.0302 (0.0201)	0.0068 (0.0055)	8 (3)	0 (0)	39 (10)	19 (10)
11. Florianópolis × **Bahia **	0.8353 (0.8345)	0.000 (0.000)	0.1197 (0.0625)	0.1000 (0.0522)	6 (4)	27 (7)	52 (14)	17 (5)
12. Juquitiba × **Bahia**	0.8403 (0.7874)	0.000 (0.000)	0.1212 (0.0656)	0.1019 (0.0516)	2 (1)	30 (8)	45 (19)	21 (8)
13. Cananéia × **Bahia**	0.8506 (0.8703)	0.000 (0.000)	0.1211 (0.0626)	0.1030 (0.0545)	1 (0)	29 (8)	46 (13)	22 (9)
14. Santa Teresa × **Bahia**	0.8624 (0.7926)	0.000 (0.000)	0.1187 (0.0685)	0.1024 (0.0543)	3 (2)	32 (9)	29 (14)	20 (7)
15. Itatiaia × **Bahia**	0.8761 (0.8020)	0.000 (0.000)	0.1130 (0.0617)	0.0990 (0.0495)	3 (2)	30 (8)	24 (11)	20 (7)
16. *An. cruzii × **Bahia**	0.8370 (0.7935)	0.000 (0.000)	0.1187 (0.037)	0.0993 (0.0505)	8 (5)	25 (6)	107 (39)	15 (4)

*F*_*ST*_, pair-wise estimates of population differentiation. *P*-value, significance of *F*_*ST *_values (evaluated by 1,000 random permutations). *Dxy*, average number of nucleotide substitutions per site between populations [49]; *Da*, number of net nucleotide substitutions per site between populations [49]. *S*1, number of polymorphic sites exclusive to the first population shown in the first column. *S*2, number of polymorphic sites exclusive to the second population shown in the first column. *Ss*, number of shared polymorphisms between the two populations. *Sf*, number of fixed differences between the two populations. The values in parentheses were calculated using only coding regions of the *timeless *gene fragment. *An. cruzii: all populations from south and south-east Brazil together without Bahia population.

**Table 4:** Non-synonymous changes on the *timeless *gene fragment

**Polymorphic Sites:**			
Site Position:	Individuals:	Codon:	Amino acid:

05 (first codon base)	Individuals from all populations analysed	**C**CC	**Pro**line
	Can03a	**T**CC	**Ser**ine
06 (second codon base)	Individuals from all populations analysed	C**C**C	**Pro**line
	Juq66a; Juq66b; Can06b; Can12b	C**T**C	**Leu**cine
08 (first codon base)	Individuals from south and south-east populations	**G**CG	**Ala**nine
	All individuals from Bahia population and Flo37a; Can02b	**A**CG	**Thr**eonine
18 (second codon base)	All individuals from south and south-east populations and Bahia19a; Bahia33a; Bahia20b	C**A**G	**Gl**utami**n**e
	Individuals from Bahia population	C**T**G	**Leu**cine

**Fixed Differences:**			

Site Position:	Individuals:	Codon:	Amino acid:

11 (first codon base)	All individuals from Florianópolis, Cananéia, Juquitiba, Itatiaia and Santa Teresa populations	**C**CG	**Pro**line
	All individuals from Bahia population	**T**CG	**Ser**ine
188 (first codon base)	All individuals from Florianópolis, Cananéia, Juquitiba, Itatiaia and Santa Teresa populations	**A**CG	**Thr**eonine
	All individuals from Bahia population	**T**CG	**Ser**ine
275 (first codon base)	All individuals from Florianópolis, Cananéia, Juquitiba, Itatiaia and Santa Teresa populations	**T**CC	**Ser**ine
	All individuals from Bahia population	**A**CC	**Thr**eonine

List of non-synonymous changes on the studied *timeless *gene fragment between *An. cruzii *populations. Flo: Florianópolis population; Can: Cananéia population; Juq: Juquitiba population; Ita: Itatiaia population; San: Santa Teresa population; Bahia: Bahia population.

The values using only coding regions (shown in parentheses in Table 3) show some differences compared with those obtained with the whole sequence. Yet even using the more conserved coding regions, the values of differentiation between the population from Bahia and all others revealed a high number of fixed differences and only a few shared polymorphisms. Among the southern and south-eastern populations, there were shared polymorphisms and no fixed differences, suggesting they belong to the same or to very closely related species.

### Divergence time between *An. cruzii *populations

The estimate of the time of divergence between *An. cruzii *populations from Bahia and the others were calculated using the *Da *value based on the third codon positions. This estimate assumed that substitutions rates observed between *An. cruzii *from Bahia State and the other populations originally from southern regions of Brazil are similar to the estimated rates in the same fragment of the *timeless *gene between closely related *Drosophila persimilis *and *Drosophila pseudoobscura*, species that diverged around 0.85 millions of years ago (MYA) (FlyBase Accession Numbers FBtr0185090 and FBtr0282161, respectively) [42]. The divergence observed for the *timeless *gene between these two *Drosophila *species based on the third codon positions is 0.03030. Based on the *Da *value (0.05426), the estimated time of divergence between *An. cruzii *populations from south and south-east Brazil and that from Bahia State, is approximately 1.5 MYA.

### Genealogy of the *An. cruzii *timeless sequences

**Figure 3:**
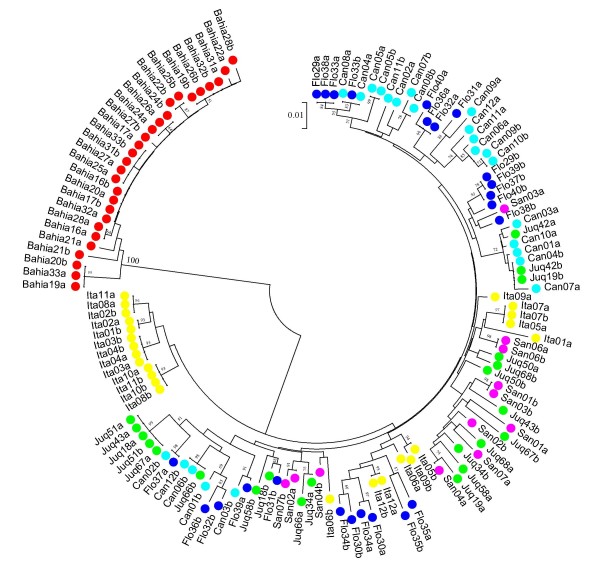
**Neighbour-joining tree**. Neighbour-joining tree using *timeless *nucleotide sequences of the *Anopheles cruzii *populations obtained with Kimura 2-parameters distance. Numbers on the nodes represent the percentage bootstrap values based on 1,000 replications. Flo: Florianópolis population; Can: Cananéia; Juq: Juquitiba; Ita: Itatiaia; San: Santa Teresa; Bahia: Itaparica Island population.

Figure 3 shows a Neighbour-joining tree of the sequences from all *An. cruzii *populations using the Kimura 2-parameter distance and the *timeless *gene sequences. The resulting tree showed no clear separation between the sequences of the populations from Florianópolis, Cananéia, Juquitiba, Itatiaia and Santa Teresa, but some differentiation was evident since the sequences do not appear at random in the tree, especially in the case of Itatiaia. The *An. cruzii *sequences from the Bahia population, however, were clearly separated on an isolated branch.

## Discussion

Zavortink [4] pointed out morphological differences in the larval stage of populations of *An. cruzii *from Rio de Janeiro and Santa Catarina States, suggesting that *An. cruzii *could represent more than a single species. A moderately high *F*_*ST *_value between Florianópolis (Santa Catarina State) and Itatiaia (Rio de Janeiro State) populations was reported here. In addition, comparison of Itatiaia with the other populations (excluding Bahia) revealed even higher *F*_*ST *_values, perhaps suggesting that this population is indeed in a process of differentiation and incipient speciation. Moreover, sequences from Itatiaia showed some clustering in the Neighbour-joining tree (Figure 3). Itatiaia was also the least polymorphic population of south and south-east Brazil and showed the highest number of homozygotes suggesting some inbreeding. It is possible that this reflects a smaller effective size and the relative isolation of this population, since its location in a valley between two mountain chains (Serra do Mar and Serra da Mantiqueira – Figure 1) might reduce gene flow with other populations.

In a recent review, Ayala and Coluzzi [43] argue that many siblings are outcomes of recent speciation processes associated with paracentric inversions, mostly involving the *X *chromosome. Ramirez and Dessen [23,24], studying the *X *chromosome banding patterns and inversion frequencies of distinct populations of *An. cruzii *from south and south-east Brazil, showed that there are three *X *chromosomal forms (A, B and C), suggesting a process of incipient speciation acting on *An. cruzii *populations. Among the localities analysed in this study, only Juquitiba and Cananéia were also investigated by Ramirez and Dessen [23,24]. They observed that in Juquitiba the majority of mosquitoes had form A and the remainder had form C, while in Cananéia form B predominated with the remainder having form A [23,24]. Although the differentiation in the *timeless *gene between these two populations is not high, the *F*_*ST *_value is significant and does not contradict the results of the chromosomal analysis. The relatively low differentiation in *timeless *among most populations from south and south-east Brazil might reflect introgression at this locus. It would be interesting to analyse the same populations with an *X*-linked molecular marker to see whether a higher level of differentiation is found.

Recently, Malafronte *et al *[26] compared sequences of ITS2 (Internal Spacer Region 2) from several *An. cruzii *populations from south and south-east Brazil. Although, they found some differences between sequences from different localities, including Juquitiba and Cananéia, they considered premature to conclude based on their results that there are distinct sibling species in the areas they investigated. Similar results were observed by Calado *et al *[44] using PCR-RAPD and PCR-RFLP of the ITS2 region.

Very strong evidence was presented here that confirms the existence of a different species in Bahia State, a finding that supports a previous isoenzyme study [25]. The extremely high *F*_*ST *_values detected between this population and the other five populations studied, as well as the higher number of fixed differences observed, show that Bahia represents a different species. This population also presented lower levels of variability than those from south and south-east Brazil, possibly indicating a smaller population size or past founder effects. However, although the isoenzyme heterozygosity reported for Bahia is lower than Cananéia it is similar to that observed in Florianópolis [25].

A very rough estimate suggests that the divergence between the Bahia population and the more southern populations of *An. cruzii *possibly occurred around 1.5 MYA, during the Pleistocene. Climate changes during this period such as an intense precipitation decrease and more arid conditions fragmented the Brazilian Atlantic forest [45] creating refugia that played an important role in the differentiation among populations of a number of forest species, such as marmosets [46], tree frogs and many others [47]. Forest fragmentation has also been proposed to explain differentiation among populations of the Atlantic forest mosquito *Sabethes albiprivus *[48]. Since *An. cruzii *is also a forest-obligate species, it is possible that the Bahia and southern populations of this species complex suffered fragmentation due a constriction of the forest. Although Tajima's *D *and Fu & Li's *D *and *F *statistics were non-significant, they were negative in most cases and that is consistent with population expansion following the forest recovery after the Pleistocene. Analysis of a number of other molecular markers will allow more precise estimates of the divergence time between the Bahia population and those of south and south-east Brazil. It may also help in determining whether further *An. cruzii *siblings exist in the latter area.

Finally, although malaria cases are reported annually in Bahia State, the main vector implicated in *Plasmodium *spp. transmission in this area is *An. darlingi *and not *An. cruzii*, the most important vector in the southern states. This suggests that the differentiation observed within the *An. cruzii *complex might also explain aspects of the vectorial capacity of these mosquitoes, however further studies are needed to confirm or reject this hypothesis.

## Conclusion

Analysis of the molecular polymorphism and genetic differentiation of the *timeless *gene among Brazilian populations of *An. cruzii *indicates that this malaria vector is a complex of at least two cryptic species, one occurring in the north-east (Bahia State) and another in south and south-east Brazil. In addition, the data also suggest that populations of the latter region might also constitute different incipient species and that further work might support the occurrence of other siblings within this complex in Brazil.

## Competing interests

The authors declare that they have no competing interests.

## Authors' contributions

LDPR participated in data generation and analysis, and drafted the manuscript. She also helped capture mosquitoes in Florianópolis. CJCP carried out the capture and morphological identification of mosquitoes collected in Florianópolis and Itaparica. CG participated in the cloning of *An. cruzii timeless *gene fragments. ECG participated in the DNA sequencing and helped to write the manuscript. AAP is the principal investigator, participated in its design and coordination, and helped to write the manuscript. All authors read and approved the final manuscript.

## Supplementary Material

Additional file 1 **Alignment of the DNA sequences of *An. cruzii***. Alignment of the DNA sequences from the *timeless *gene fragment from all populations of *An. cruzii *analysed. The translated amino acid sequence is shown above the alignment and the introns are presented in the darkened regions. Dots represent the identity of the first nucleotide sequence and asterisks represent the identity of all sequences. The non-synonymous changes found among the sequences are highlighted in yellow boxes. Flo: individuals from Florianópolis; Can: Cananéia; Juq: Juquitiba; Ita: Itatiaia; San: Santa Teresa; Bahia: individuals from Itaparica Island, Bahia State. Click here for file
