# Supplementary material for: Assessing the molecular divergence between Anopheles (Kerteszia) cruzii populations from Brazil using the timeless gene: further evidence of a species complex
Source: Malar J. 2009 Apr 9;8:60. doi: 10.1186/1475-2875-8-60 (PMC2673228; doi:10.1186/1475-2875-8-60)
Supplement: Additional file 1 — Alignment of the DNA sequences of An. cruzii. Alignment of the DNA sequences from the timeless gene fragment from all populations of An. cruzii analysed. The translated amino acid sequence is shown above the alignment and the introns are presented in the darkened regions. Dots represent the identity of the first nucleotide sequence and asterisks represent the identity of all sequences. The non-synonymous changes found among the sequences are highlighted in yellow boxes. Flo: individuals from Florianópolis; Can: Cananéia; Juq: Juquitiba; Ita: Itatiaia; San: Santa Teresa; Bahia: individuals from Itaparica Island, Bahia State. [file 1475-2875-8-60-S1.doc]

00000000000000000000000000000000000000000000000000000000000000000000000000000000000000000000000000011111111111111111111111111111111111111111111111111111111111111111111111111111111111111111111111111112222222222222222222222222222222222222222222222222222222222222222222222222222222222222222222222222222333333333333333333333333333333333333333333333333333333333333333333333333333333333333333333333333333344444444444444444

00000000011111111112222222222333333333344444444445555555555566666666677777777778888888888999999999900000000001111111111222222222233333333334444444444555555555566666666667777777777888888888899999999990000000000111111111122222222223333333333444444444455555555556666666666777777777788888888889999999999000000000011111111112222222222333333333344444444445555555555666666666677777777778888888888999999999900000000001111111

12345678901234567890123456789012345678901234567890123456789012345678901234567890123456789012345678901234567890123456789012345678901234567890123456789012345678901234567890123456789012345678901234567890123456789012345678901234567890123456789012345678901234567890123456789012345678901234567890123456789012345678901234567890123456789012345678901234567890123456789012345678901234567890123456789012345678901234567890123456

N P A P A Q E K K E L R R K K L V K R G K S N I I N M K G L M H H V P T D D D I S H I L K E F T V D F L L K G Y G Y L V Q E L H S Q L L S D L

Flo32a CAATCCCGCGCCGGCGCAGGAGAAGAAGGAACTGCGGCGCAAGAAGCTGGTGAAGCGGGGCAAGAGCAACATGTCAGTAGGAACCGGAAAGGGCGCCCCAGTCCCGGCTCGTTCACCGCAACTGAGA-TTT-GTTTTCCAACCTTTTC--CAGCATCAATATGAAGGGCCTGATGCACCACGTCCCGACGGACGATGACATATCGCATATCCTGAAGGAGTTCACGGTTGACTTTCTGCTCAAAGGCTACGGCTATCTGGTGCAGGAGCTGCACTCCCAGTTGCTGTCGGATTTGGTGAGTCCTGGTCCGATGGTTCACTTTCCTGCGGTTTGTGGAGAAG-GTACCCTGTGACGGTTTTTA--ATCATTCCG--TTCCAATCCCTCTTTCG-GACGTTCCGACTTTATCGACAGC

Flo29a ..................A.............................................................................T......G.......................-...-................--...............................................................................................................................................................................................-.........TC....A....--.........--...G............C-.................C.....

Flo29b ................................................................................................T...A..G...................A...-...-...............--C.......................................................................................................................................................................................C.......-..........C.........--.........--...G...A........C-.................C.....

Flo30a ..............TT....................A...........................................................T.....................T........-...-...............TT-.................................T...........................................................................................................................................-................T-.......TC.C.....C...--.........--...G............C-.G.....--........C.....

Flo30b ...............T................................................................T...............T.....................T........-...-...............T-C.................................T............................................................................................................................................................T-........C.C.....C...--..T......--...G.....T......C-............C....C.....

Flo31a ................................................................................................T......G.......................-...-................--..............................................................................C..................................................................................................A.......A....T-....................--...G.....--...G........C...T-.G.....--........C.....

Flo31b ......................................................A.........................................T......G.......................-...-...............--C..............................................................................C.............................................................................................T.................T-..........C.........--..A......--...G....T.......C-........A........C.....

Flo32b ...........................A....................................................................T......G.......................-...-...............--C..................T.................A...................................................................................................................G..............................C.......-A.........C......C..--..A......--..T.............C-.................C.....

Flo33a ..................A.............................................................................T......G.......................-...-................--...............................................................................................................................................................................................-.........TC....A....--.........--...G............C-.................C.....

Flo33b ..................A............................................................................................................-...-................--...............................................................................................................................................................................................-.........TC....A....--.........--...G............C-.................C.....

Flo34a ...............C....................A...........................................................T.....................T........-...-...............TT-.................................T...........................................................................................................................................-................T-.......TC.C.....C...--.........--...G............C-.G.....--........C.....

Flo34b ...............C..A.............................................................................T..............................-...-...............--C.................................T............................................................................................................................................................T-........C.C.....C...--..T......--...G.....T......C-............C....C.....

Flo35a ...............C..A.................A...........................................................T..............................-...-...............--C.................................T.............................................................................................................................................C...........A...-......G...C.....C...--.........--................C-..........T......C.....

Flo35b ...............C..A.............................................................A...............T.T............................-...-...............--C..................T..............T....................................................................................T........................................................C...........A...-..........C.....C...--.........--...G.....T......C-............C....C.....

Flo36a ...............................................................................................................................-...-................--...............................................................................................................................................................................................-....................--.........--.................-.......................

Flo36b .........A........A.............................................................................T......G.......................-...-...............--C..................T.................A..............................................................T....................................................G..............................C.......-A.........C......C..--..A......--..T.............C-.................C.....

Flo37a .......A........................................................................................T..............................-...-...............--C.................................................................................T.....................................................................................................C.......-..........C......C..--..A......--..T...C.........C-.................C.....

Flo37b ................................................................................................T...A..G...................A...-...-...............--C.......................................................................................................................................................................................C.......-..........C.........--.........--...G............C-.................C.....

Flo38a ..................A.............................................................................T......G.......................-...-................--...............................................................................................................................................................................................-.........TC....A....--.........--...G............C-.................C.....

Flo38b ...........................A....................................................................T......G.......................-...-...............--C..................................................................................................................................................................A.........T.................T-..........C.........--.........--...G............C-.................C.....

Flo39a .........................................................................................C......T..............................-...-...............--C..................T...........................................................C.............................................................................................T.................T-..........C......C..--..A......--..T.............C-.................C.....

Flo39b ................................................................................................T...A..G...................A...-...-...............--C.......................................................................................................................................................................................C.......-..........C.........--.........--...G...A........C-.................C.....

Flo40a ...............................................................................................................................-...-................--........................................................................................................CC.....................................................................................-....................--.........--.................-.......................

Flo40b ...........................A....................................................................T......G.......................-...-...............--C.......................................................................................................................................................................................C.......-..........C.........--..A......--...G............C-.................C.....

Can01a ..................A.............................................................................T......G.......................-...-................--..............................................................................C........................................................................................................C.A.....-..........C...G.....--.........--...G............C-..........T......C.....

Can01b ................................................................................................T......G.......................-...-...............--C..................T.................A...................................................................................................................G...................T..........C.......-A.........C......C..--..A......--...G....T.......C-........A........C.....

Can02a ..................A............................................................................................................-...-................--..............................................................................G........................................................................................................C.......-........C.C.........--.........--...G............C-.G...............C.....

Can02b .......A........................................................................................T..............................-...-...............--C.................................................................................T.....................................................................................................C.......-..........C......C..--..A......--..T...C.........C-.................C.....

Can03a ....T...........................................................................................T......G.......................-...-................--.......................................................................................................................................................................................C.A.....-..........C...G.....--.........--...G............C-..........T......C.....

Can03b .........................................................................................C......T......G.......................-...-...............--C..................T...........................................................C......T......................................................................................T.................T-..........C......C..--..A......--..T.............C-.................C.....

Can04a ..................A............................................................................................................-...-................--...............................................................................................................................................................................................-.........TC....A....--.........--...G............C-.................C.....

Can04b ..................A.............................................................................T......G.......................-...-................--.......................................................................................................................................................................................C.A.....-..........C...G.....--.........--...G............C-..........T......C.....

Can05a ..................A.............................................................................A..........................T...-...-................--.....................A......................................................................................................................................................T.................T-..........C....A....--.........--...G............C-.................C.....

Can05b ..................A.............................................................................A..........................T...-...-................--.....................A......................................................................................................................................................T.................T-..........C....A....--.........--...G............C-.................C.....

Can06a ..................A...............................................................................-............................-...-................--.........................................................................................................................................................................................A....T-....................--.........--................C-........C.T......C.....

Can06b .....T..........................................................................................T.............................G-...T......-........--C........................................................................................................................................................G...................T..........C.......-..........C......C..--..A......--...G....T.......C-........A........C.....

Can07a ..................A..........................A..............................................A...T......G.......................-...-................--.......................................................................................................................................................................................C.A.....-..........C...G.....--.........--...G............C-..........T......C.....

Can07b ..................A..........................A..................................................T................T............G-...T................--.......................................................................................................................................................................................C.......-........C.C.........--.........--...G............C-.G...............C.....

Can08a ..................A............................................................................................................-...-................--.....................................................................................................................................................................-.........................-.........TC....A....--.........--...G............C-.................C.....

Can08b ..................A..........................A..................................................T................T............G-...T................--.......................................................................................................................................................................................C.......-..........C.........--.........--...G............C-.................C.....

Can09a ..................A.....................................................................................AT.....................-...-................--..............................................................................C..........................................................................................................A....T-....................--...G.....--...G........C...T-.G.....--........C.....

Can09b ..................A............................................................................................................-...-................--.........................................................................................................................................................................................A....T-....................--.........--................C-...T......T......C.....

Can10a ................................................................................................T......G.......................-...-................--..............................................................................C........................................................................................................C.A.....-..........C...G.....--.........--...G............C-..........T......C.....

Can10b ..................A............................................................................................................-...-................--.........................................................................................................................................................................................A....T-....................--.........--................C-...T......T......C.....

Can11a ..................A..............................................................................................T.............-...-................--.........................................................................................................................................................................................A....T-....................--.........--................C-..........T......C.....

Can11b ......................................................A........................................................................-...-................--..................T.................A..................................................................................................................................................C.......-..........C.........--.........--...G............C-.................C.....

Can12a ..................A........................................................................C....T......G.......................-...-............C...--.........................................................................................................................................................................................A....T-....................--.........--................C-..........T......C.....

Can12b .....T..........................................................................................T.............................G-...T...............--C........................................................................................................................................................G...................T..........C.......-..........C......C..--..A......--...G....T.......C-........A........C.....

Juq18a ............A...................................................................................T......G.......................-...-...............--C............................................................T..........................................................................................................................C.......-..........C......C..--..A......--..T............TC-.................C.....

Juq18b ...............T................................................................................T......G.......................-...-...............--C..............................................................................C.............................................................................................T.................T-..........C.........--..A......--...G....T.......C-........A........C.....

Juq19a ...............T................................................................................T..............................-...-........T.......--.....................................................................A........C..T..C...................................A.....T.............................................T..................-...................TT-..A......--...G............C-.................C.....

Juq19b ..................A.............................................................................T......G.......................-...-................--.......................................................................................................................................................................................C.A.....-..........C...G.....--.........--...G............C-..........T......C.....

Juq34a ...............T...............................................................................AT..............................-...-...............--C..............................................................................C.......................................T..................................................................A.....-..........C.........--..A......--...G............C-.................T.....

Juq34b ...............T................................................................................T..............................-...-........T......--C.....................................................................A........C..T..C................................A.........................................................................-..........C.........--.........--...G............C-.................C.....

Juq42a ...............T................................................................................T......G.......................-...-................--.......................................................................................................................................................................................C.A.....-..........C...G.....--.........--...G............C-..........T......C.....

Juq42b ..................A.............................................................................T......G.......................-...-................--.......................................................................................................................................................................................C.A.....-..........C...G.....--.........--...G............C-..........T......C.....

Juq43a ............A...................................................................................T......G.......................-...-...............--C............................................................T..........................................................................................................................C.......-..........C......C..--..A......--..T............TC-.................C.....

Juq43b .........A........A..................................................................A..........T......G.......................-...-...............--C.....................................................................A........C..T..C...........................................................................................T......C.......-..........C.........--.........--...G............C-.................C.....

Juq50a ...............T.....A....................................................................................A....................-...-...............--C..............................................................................C......................................................................................C......T..................-..........C.........--.........--...G............C-.................C.....

Juq50b ............A........................................................................A..........T......G.......................-...-...............--C..............................................................................C.............................................................................................T..................-..........C.........--.........--...G............C-.................C.....

Juq51a ............A...................................................................................T......G.......................-...-...............--C............................................................T..........................................................................................................................C.......-..........C......C..--..A......--..T............TC-.................C.....

Juq51b ............A...................................................................................T......G.......................-...-...............--C............................................................T..........................................................................................................................C.......-..........C......C..--..A......--..T............TC-.................C.....

Juq58a .........A..........................A...........................................................T..............................-...-........T......--C.....................................................................A........C..T..C...........T...........................................................................T..................-..........C.........--..A......--...G...G........C-.................C.....

Juq58b ...............................................................................................................................-...-...............--C..................T...........................................................C..................................................................................C..........T..A..............T-..........C.........--..A......--...G....T.......C-........A........C.....

Juq66a .....T.........T..............................................................T................AT..............................-...-...............--C..............................................................................C.......................................T..................................................................A.....-..........C.........--..A......--...G...G........C-.................C.....

Juq66b .....T..........................................................................................T.............................G-...T...............--C........................................................................................................................................................G...................T..........C.......-..........C......C..--..A......--...G....T.......C-........A........C.....

Juq67a ............A...................................................................................T......G.......................-...-...............--C............................................................T..........................................................................................................................C.......-..........C......C..--..A......--..T............TC-.................C.....

Juq67b ................................................................................................T......G..A....................-...-...............--C.............................................................T.......A........C..T..C..........................................................................................................-..........C......C..--..A......--..T.............C-......T..........C.....

Juq68a ...............T................................................................................T....................A.........-...-........T......--C..................T...........................................................C..T..C.........................................T...........A....................................................-..........C.........--.........--...G............C-.................C.....

Juq68b .....................................................................................A....................A....................-...-...............--C..............................................................................C.............................................................................................T..................-..........C.........--.........--...G...G........C-.................C.....

Ita01a ..................A................................A............................................T......G..A..................C.-...-...............--C..................T...........................................................C........................C...........................................................A........-.................T-..........C.........--.........--...G.....T......C-.................C.....

Ita01b ..................A.............................................................................T.....................T........-...-...............T-C.................................T...........C................................C.............................................................................................T.................T-..........C.....C...--.........--...G............C-.................C.....

Ita02a ..................A.............................................................................T.....................T........-...-...............T-C.................................T...........C................................C.............................................................................................T.................T-..........C.....C...--.........--...G............C-.................C.....

Ita02b ..................A.............................................................................T.....................T........-...-...............T-C.................................T...........C................................C........................................................................T.------.............T..................-..........C.........--..A......--...G............C-.................C.....

Ita03a ...............T.....A..........................................................................T.....................T........-...-...............T-C.................................T...........C................................C.............................................................................................T.................T-..........C.........--.........--...G............C-.................C.....

Ita03b ...............T.....A..........................................................................T.....................T........-...-...............T-C.................................T...........C................................C.............................................................................................T.................T-..........C.........--.........--...G............C-.................C.....

Ita04a ...............T.....A..........................................................................T.....................T........-...-...............T-C.................................T...........C................................C.............................................................................................T.................T-..........C.........--.........--...G............C-.................C.....

Ita04b ...............T.....A..........................................................................T.....................T........-...-...............T-C.................................T...........C................................C.............................................................................................T.................T-..........C.........--.........--...G............C-.................C.....

Ita05a ...............T................................................................................T......G..A..................C.-...-...............--C.................................T............................................C.............................................................................................T..................-..........C.........--.........--...G............C-.................C.....

Ita05b ...............C..A............................................A................................T..............................-...-...............--C..............................................................................C.............................................................................................T..................-..........C.........--.........--...G............C-........T........C.....

Ita06a ...............C..A.............................................................................T..............................-...-...............T-C.................................T............................................C.............................................................................................T.................T-..........C.........--.........--...G............C-.................C.....

Ita06b .........A......................................................................A...............T..............................-...-...............TT-..................T...........................................................C.............................................................................................T.................T-..........C.........--.........--...A........C...C-.G.....--........C.....

Ita07a ...............T................................................................................T......G..A..................C.-...-...............--C.................................T............................................C.............................................................................................T..................-..........C.........--.........--...G............C-.................C.....

Ita07b ...............T................................................................................T......G..A..................C.-...-...............--C.................................T............................................C.............................................................................................T..................-..........C.........--.........--...G............C-.................C.....

Ita08a ..................A.............................................................................T.....................T........-...-...............T-C.................................T...........C................................C........................................................................T.------.............T..................-..........C.........--..A......--...G............C-.................C.....

Ita08b ..................A.............................................................................T.....................T........-...-...............--C..................T..........................C..............................................................................................................................T..................-..........C.........--..A......--...G............C-.................C.....

Ita09a .........A......................................................................................T..............................-...-...............--C..............................................................................C.............................................................................................T..................-..........C.........--.........--...G............C-........T........C.....

Ita09b ...............C..A............................................A................................T..............................-...-...............--C...........................T..................................................C.............................................................................................T..................-..........C.........--.........--...G............C-........T........C.....

Ita10a ..................A.............................................................................T.....................T........-...-...............--C..................T..........................C..............................................................................................................................T..................-..........C.........--..A......--...G............C-.................C.....

Ita10b ..................A.............................................................................T.....................T........-...-...............--C..................T..........................C..............................................................................................................................T..................-..........C.........--..A......--...G............C-.................C.....

Ita11a ..................A.............................................................................T.....................T........-...-...............T-C.................................T...........C................................C........................................................................T.------.............T..................-..........C.........--..A......--...G............C-.................C.....

Ita11b ..................A.............................................................................T.....................T........-...-...............--C..................T..........................C..............................................................................................................................T..................-..........C.........--..A......--...G............C-.................C.....

Ita12a ...............T.....A..........................................................................T..............................-...-...............--C.................................T..........................................................................................................................................T.................T-..........C.....C...--.........--...G............C-.................C.....

Ita12b ...............T.....A..........................................................................T..............................-...-...............--C.................................T..........................................................................................................................................T.................T-..........C.....C...--.........--...G............C-.................C.....

San01a ...............T.....................................................................A....................A.A.......G..........-...-...............--C.....................................................................A........C..T..C................................................T......................................T..................-..........C.........--..A......--...G............C-.................C.....

San01b ................................................................................................T......G.......................-...-...............--C...................T.................................................A........C..T..C.......................................................................................T.................T-..........C.........--.........--...G............C-................TC.....

San02a ...............T...............................................................................TT..............................-...-...............--C..................T...........................................................C.............................................................................................T..................-..........C.........--..A......--...G...G........C-.................C.....

San02b ...............T................................................................................T..............................-...-...............--C.....................................................................A........C..T..C.......................................................................................T..................-..........C.........--.........--...G....T.......CC.................T.....

San03a ...........................A....................................................................T......G.T.....................-...-...............--C..................G............................................................................................................................................................................-..........C.........--.........--...G...G........C-.................C.....

San03b ................................................................................................T......G.......................-...-...............--C...................T.................................................A........C..T..C.......................................................................................T.................T-..........C.........--.........--...G............C-................TC.....

San04a ................................................................................................T..............................-...-........T.......--.....................................................................A........C..T..C..........................................................................................................-...................TTT..A......--...G............C-.................C.....

San04b ...............T................................................................................T..............................-...-...............--C..................T...........................................................C...............................................................................................................T-..........C.........--.........--...G............C-.................C.....

San06a .........A........A..........................................................................T..T......G..A....................-...-...............--C..............................................................................C.............................................................................................T..................-..........C.........--.........--...G............C-.................C.....

San06b .........A........A..........................................................................T..T......G..A....................-...-...............--C..............................................................................C.............................................................................................T..................-..........C.........--.........--...G............C-.................C.....

San07a ...............T................................................................................T..............................-...-...............--C.................................T...................................A........C..T..C.........................................T...........A....................................................-..........C.........--T........--...G...G........C-.................C.....

San07b ...............T..............................T.................................................T..............................-...-...............--C..................T...........................................................C.............................................................................................T..................-..........C.........--..A......--...G...G........C-.................C.....

Bahia16a .......A..T....C.T............G..........................................................CC...--.G.GT..G.A.T...C.GGTA.T.....GT.G...T.A.........C....TC.........C...........................T.......C................................C.......................................T.....A.....C........................------....-------....T.............AT.CC.......C..T.G....--..T.....CCC...G......C.....C-.G.....--.......--.....

Bahia16b .......A..T....C.T............G..........................................................CC...--.G.GT..G.A.T...C.GGTA.T.....GT.G...C.A.........C....TC.........C...........................T.......C................................C.......................................T.....A.....C........................------....-------....T.............AT.CC.......C..T.G....--........CCC...G......C.....C-.G.....--.......--.....

Bahia17a .......A..T....C.T............G..........................................................CC...--.G.GT..G.A.T...C.GGTA.T.....GT.G...C.A.........C....TC.........C...........................T.......C................................C.......................................T.....A.....C........................------....-------....T.............AT.CC.......C..T.G....--........CCT...G......C.....C-.G.....--.....T.--.....

Bahia17b .......A..T....C.T............G..........................................................CC...--.G.GT..G.A.T...C.GGTA.T.....GT.G...C.A.........C....TC.........C...........................T.......C................................C.......................................T.....A.....C........................------....-------....T.............AT.CC.......C..T.G....--........CCC...G......C.....C-.G.....--.......--.....

Bahia19a .......A.AT...................G..........................................................CC...--.G.GT..G.A.T...C.GGTA.T.....GT.G...C.A.........C....TC.........C...........................T.......C................................C.......................................T.....A.....C........................------....-------....T.....A.......AT.CC.......C..T.G....--........CCC...G......C.....C-.G..C..--.....T.--.....

Bahia19b .......A..T....C.T............G..........................................................CC...--.G.GT..G.A.T...C.GGTA.T.....GT.G...T.A.........C....TCT........C...........................T.......C................................C.......................................T.....A.....C........................------....-------....T.............AT.CC.......C..T.G....--........CCC...G......C.....C-.G.....--.......--.....

Bahia20a .......A..T....C.T............G..........................................................CC...--.G.GT..G.A.T...C.GGTA.T.....GT.G...C.A.........C....TC.........C...........................T.......C................................C.......................................T.....A.....C........................------....-------....T.............AT.CC.......C..T.G....--........CCC...G......C.....C-.G.....--.......--.....

Bahia20b .......A.AT...................G..........................................................CC...--.G.GT..G.A.T...C.GGTA.T.....GT.G...C.A.........C....TC.........C...........................T.......C................................C.......................................T.....A.....C........................------....-------....T.....A.......AT.CC.......C..T.G....--........CCC...G......C.....C-.G..C..--.....T.--.....

Bahia21a .......A..T....C.T............G..........................................................CC...--.G.GT..G.A.T...C.G.TA.T.....GT.G...C.A.........C....TC.........C...........................T.......C................................C....................T..................T.....A.....C........................------....-------....T.....T.......AT.CC.......C..T.G....--........CCC...G......C.....C-.G.....--.......--.....

Bahia21b ......TA..T....C.T............G..........................................................CC...--.G.GT..G.A.T...C.GGTA.T.....GT.G...C.A.........C....TC.........C...........................T.......C....................A...........C.......................................T.....A.....C................... .....------....-------....T..............T.CT.......C..T.G....--........CCC...A......C.....C-.G.....--.......--....

Bahia22a .......A..T....C.T............G..........................................................CC...--.G.GT..G.A.T...C.GGTA.T.....GT.G...T.A.........C....TCT........C.........T.................T.......C................................C.......................................T.....A.....C........................------....-------....T.............AT.CC.......C..T.G....--........CCC...G......C.....C-.G.....--.......--.....

Bahia22b .......A..T....C.T............G..........................................................CC...--.G.GT..G.A.T...C.GGTA.T.....GT.G...C.A.........C....TC.........C...........................T....T..C................................C.......................................T.....A.....C........................------....-------....T.............AT.CC..T....C..T.G....--........CCC...G......C.....C-.G.....--.......--.....

Bahia24a .......A..T....C.T............G..........................................................CC...--.G.GT..G.A.T...C.GGTA.T.....GT.G...C.A.........C....TC.........C...........................T.......C................................C..........................C............T.....A.....C........................------....-------....T.............AT.CC.......C..T.G....--........CCC...G......C.....C-.G.....--.......--.....

Bahia24b .......A..T....C.T............G..........................................................CC...--.G.GT..G.A.T...C.GGTA.T.....GT.G...C.A.........C....TC.........C...........................T.......C................................C..........................C............T.....A.....C........................------....-------....T.............AT.CC.......C..T.G....--........CCC...G......C.....C-.G.....--.......--.....

Bahia25a .......A..T....C.T............G..........................................................CC...--.G.GT..G.A.T...C.GGTA.T.....GT.G...C.A.........C....TC.........C...........................T.......C................................C.......................................T.....A.....C........................------....-------....T.............AT.CC.......C..T.G....--........CCT...G......C.....C-.G.....--.....T.--.....

Bahia25b .......A..T....C.T............G..........................................................CC...--.G.GT..G.A.T...C.GGTA.T.....GT.G...C.A.........C....TC.........C...........................T....T..C................................C.......................................T.....A.....C........................------....-------....T.............AT.CC..T....C..T.G....--........CCC...G......C.....C-.G.....--.......--.....

Bahia26a .......A..T....C.T............G..........................................................CC...--.G.GT..G.A.T...C.GGTA.T.....GT.G...C.A.........C....TC.........C...........................T.......C................................C..........................C............T.....A.....C........................------....-------....T.............AT.CC.......C..T.G....--........CCC...G......C.....C-.G.....--.......--.....

Bahia26b .......A..T....C.T............G..........................................................CC...--.GTGT..G.A.T...C.GGTA.T.....GT.G...T.A.........C....TCT........C...........................T.......C................................C.......................................T.....A.....C........................------....-------....T.............AT.CC.......C..T.G....--........CCC...G......C.....C-.G.....--.......--.....

Bahia27a .......A..T....C.T............G..........................................................CC...--.G.GT..G.A.T...C.GGTA.T.....GT.G...C.A.........C....TC.........C...........................T.......C................................C.......................................T.....A.....C........................------....-------....T.............AT.CC.......C..T.G....--........CCT...G......C.....C-.G.....--.....T.--.....

Bahia27b A......A..T....C.T............G..........................................................CC...--.G.GT..G.A.T...C.GGTA.T.....GT.G...C.A.........C....TC.........C...........................T.......C................................C.......................................T.....A.....C........................------....-------....T.............AT.CC.......C..T.G....--........CCC...G......C.....C-.G.....--.......--.....

Bahia28a .......A..T....C.T............G..........................................................CC...--.G.GT..G.A.T...C.GGTA.T.....GT.G...T.A.........C....TC.........C...........................T.......C................................C.......................................T.....A.....C........................------....-------....T.............AT.CC.......C..T.G....--..T.....CCC...G......C.....C-.G.....--.......--.....

Bahia28b .......A..T....C.T............G..........................................................CC...--.G.GT..G.A.T...C.GGTA.T.....GT.G...T.A.........C....TCT........C...........................T.......C................................C.......................................T.....A.....C........................------....-------....T.....T.......AT.CC.......C..T.G....--........CCC...G....T.C.....C-.G.....--.......--.....

Bahia31a .......A..T....C.T............G..........................................................CC...--.G.GT..G.A.T...C.GGTA.T.....GT.G...T.A.........C....TCT........C...........................T.......C................................C.......................................T.....A.....C........................------....-------....T.............AT.CC.......C..T.G....--........CCC...G......C.....C-.G.....--.......--.....

Bahia31b .......A..T....C.T............G..........................................................CC...--.G.GT..G.A.T...C.GGTA.T.....GT.G...C.A.........C....TC.........C...........................T.......C................................C.......................................T.....A.....C........................------....-------....T.............AT.CC.......C..T.G....--........CCT...G......C.....C-.G.....--.....T.--.....

Bahia32a .......A..T....C.T............G..........................................................CC...--.G.GT..G.A.T...C.GGTA.T.....GT.G...C.A.........C....TC.........C...........................T.......C................................C.......................................T.....A.....C........................------....-------....T.............AT.CC.......C..T.G....--........CCC...G......C.....C-.G.....--.......--.....

Bahia32b .......A..T....C.T............G..........................................................CC...--.GTGT..G.A.T...C.GGTA.T.....GT.G...T.A.........C....TCT........C...........................T.......C................................C.......................................T.....A.....C........................------....-------....T.............AT.CC.......C..T.G....--........CCC...G......C.....C-.G.....--.......--.....

Bahia33a .......A.AT...................G..........................................................CC...--.G.GT..G.A.T...C.GGTA.T.....GT.G...C.A.........C....TC.........C...........................T.......C................................C.......................................T.....A.....C........................------....-------....T.....A.......AT.CC.......C..T.G....--........CCC...G......C.....C-.G..C..--.....T.--.....

Bahia33b .......A..T....C.T............G..........................................................CC...--.G.GT..G.A.T...C.GGTA.T.....GT.G...C.A.........C....TC.........C...........................T.......C................................C.......................................T.....A.....C........................------....-------....T.............AT.CC.......C..T.G....--........CCT...G......C.....C-.G.....--.....T.--.....

*** * * * * ** ***** ** ***** ******** **** ** ******** ************** * **** *** ** ** * **** *** * **** * ** ** ******** ******** * ***** ***** ** **** ** ************** **** ** ******** ** ** ********** ** *** *********** * *** * *** ** **** *********** * * **** * * ** ** ** * * **** ** ** * ** * * * * * * * ******
